# ESPO AND SOCIAL RESEARCH, POLICY, AND PRACTICE SECTION SYMPOSIUM: LAW AS A DETERMINANT OF HEALTH: A SOCIAL JUSTICE LENS TO IMPACTFUL POLICY ENGAGEMENT

**DOI:** 10.1093/geroni/igad104.0157

**Published:** 2023-12-21

**Authors:** Sara Bybee, Kexin Yu, Rita Choula

## Abstract

The phrase “social determinants of health” has become a buzzword in health-related research and practice. While the US has long recognized that social determinants such as housing conditions, access to medical care, and neighborhood safety contribute to health outcomes, there has been little attention on more upstream factors. Structural, or political determinants of health are the determinants of the determinants. Political determinants of health refer to laws and policies that can inequitably distribute resources which creates structural barriers to equity for population groups that lack power and privilege. Unfortunately, many current US laws and policies perpetuate health inequities as they were originally written to protect those in power and punish those without power and privilege. Structural stigma and discrimination disproportionately affect historically marginalized and minoritized populations. Laws that disproportionately affect historically marginalized groups need to be identified, systemically tracked and revised if we aim to address health equity. In this symposium, speakers will discuss their research into the health-harming effects of laws and policies that disproportionately affect people of color and older adults. Speaker one will review GSA-related policy and advocacy activities. Speaker two will describe how to use law to address racial disparities in geographic access. Speaker three will discuss housing related laws and policies that perpetuate health inequities for older adults and disproportionately affect people from marginalized groups. This session is intended to fill a gap in current programming by making policy-related discussions more accessible and understandable for individuals who may not have experience in this area.

